# A Structured Review to Assess the Current Status of Cost-Based Burns Research in Nepal

**DOI:** 10.1093/jbcr/iraa125

**Published:** 2020-08-11

**Authors:** Julia L Lowin, Ak Narayan Poudel, Patricia E Price, Tom S Potokar

**Affiliations:** 1 Swansea Centre for Health Economics (SCHE), Swansea University; 2 Interburns, Welsh Centre for Burns and Plastic Surgery; 3 Centre for Global Burn injury Policy and Research, Swansea University, Swansea, UK

## Abstract

The management of burns is costly and complex. The problem is compounded in low and middle income countries (LMICs) where the incidence of burn injuries is high but infrastructure and funding for management and prevention is limited. Cost of illness studies allows for quantification of the costs associated with public health problems. Without cost quantification, focus and allocation of funding is challenging. The authors explored the availability of cost-focused burns research data in a target LMIC. The focus of their research was Nepal. A structured literature review including published papers, Ministry of Health (MOH) and World Health Organization (WHO) statistics was conducted to identify cost of illness studies or evidence relating to burn-related resource and costs. Gaps in the evidence base were highlighted. Research methodologies from other LMICs were reviewed. We found 32 papers related to burn injury in Nepal, one key MOH document and one relevant WHO data source. Most research focused on the epidemiology and etiology of burns in Nepal. Of the papers, only 14 reported any type of burn-related resource use and only 1 paper directly reported (limited) cost data. No studies attempted an overall quantification of the cost of burns. MOH statistics provided no additional insight into costs. Our study found an almost complete lack of cost-focused burns research in Nepal. Primary research is needed to quantify the cost of burns in Nepal. Initial focus could usefully be on the cost of care in tertiary hospitals. A full cost of burns for Nepal remains some way off.

The management of burns is costly and complex, with mortality and morbidity disproportionately affecting low and middle income countries (LMICs).^1,2^

The World Health Organization (WHO) and the International Society for Burn Injuries are developing strategies to improve the prevention and management of burn injuries, with particular focus on LMICs. Without evaluation of the resources and costs of burn care, it is challenging to make a case for funding and allocate and plan resources efficiently. Capturing and quantifying the economic consequences of burns is an important step toward a systems-wide approach to improving burn care and prevention. Robust estimates of the economic impact of burns help us to determine government and local-level priorities for intervention and funding support, whereas lack of reliable data detracts from appropriate political priority and attention.^3,4^

The Center for Global Burn Injury Policy and Research (CGBIPR) has been collaborating with local burns units in Nepal to create an integrated system for the prevention and management of burns, but a comprehensive profile of burn-related costs is currently lacking.^5^ Cost of illness studies provide a methodological framework for quantification of the economic burden of burn injuries and highlight the proportion of costs that could be saved, or spent elsewhere, through reduction in the burden (via treatment or prevention). They can provide a robust framework for public advocacy on funding for prevention, treatment, and management programmes but require robust cost data.^6^

We report an exploration of the current status of cost-focused burns research in Nepal. Our objectives were to summarize the available cost evidence and highlight the current evidence gaps that might limit a comprehensive estimate of the cost burden of burns in Nepal. A secondary objective was to analyze these evidence gaps and make preliminary recommendations on possible sources and methods to address them and enable conduct of a comprehensive cost of illness analysis. The current research was conducted in parallel to on-the-ground fieldwork focused on the feasibility of conducting primary cost collection studies in the context of a specialist care hospital (to be reported elsewhere).

## METHODS

A general literature review was conducted in MEDLINE in order to establish the extent of published data on burns in Nepal. Following this, a targeted literature review was conducted to identify studies and research which focused on cost. Our hypothesis was that cost-focused research would be limited. Our review was therefore expanded to include the Institute for Health Metrics and Evaluation (IHME) global burden of disease (GBD) platform,^7^ available Nepalese Ministry of Health (NMOH) statistics,^8^ and the WHO-CHOICE (Choosing Interventions that are Cost-Effective) database.^9^ The searches were also replicated in Google Scholar (for supplementary identification of nonacademic citations^10^).

We conducted the structured searches of published literature in October 2019. Inclusion and exclusion criteria for the studies from MEDLINE and the supplementary Google Scholar search are described below. Inclusion criteria for the NMOH and WHO databases were presented in English and providing information on at least one parameter relevant to assessment of burn-related resource or cost. All article titles were screened and checked for relevance and the reference list of each selected paper was manually screened for additional relevant references, likewise, the online database documents.

The estimate of resource and cost is a complex construct dependent on geographies (rural and urban), cost perspectives (patient, provider, and purchaser), and types of care (traditional healing, community-based, and hospital-based). Resource and expenditure is driven by multiple factors including the specific etiology of the burn (eg, type and positioning of burn), population (eg, adult vs pediatric), delivery of treatment (type of healthcare professional contact), setting of treatment (community, secondary, or tertiary hospital) methods of treatment (eg, simple dressings, pain medications, anxiolytics, surgery, reconstruction, and ongoing rehabilitation), length and type of hospitalization (eg, general ward vs ICU) travel and transportation costs, and indirect costs (days off work, disability days, and caregiver burden). We included any paper reporting a Nepalese study that might provide insight into the costs of burns without limit to perspective, setting, or indication. Note: We did not search or extract data on epidemiology, etiology, or outcomes (eg, infections, contracture, or mortality).

The proportion of research papers with a cost-focus was estimated based on initial hits. Relevant information on either resource use or expenditure was identified from the published articles and the NMOH and online databases. Evidence gaps were highlighted and analyzed against the methodology papers in order to identify possible further activities that could help guide additional primary research.

## RESULTS

The initial searches confirmed limited cost-based research on burns in Nepal, despite an anecdotally reported high burden of burns, costs, and unmet need.^11,12^

**Figure 1.**
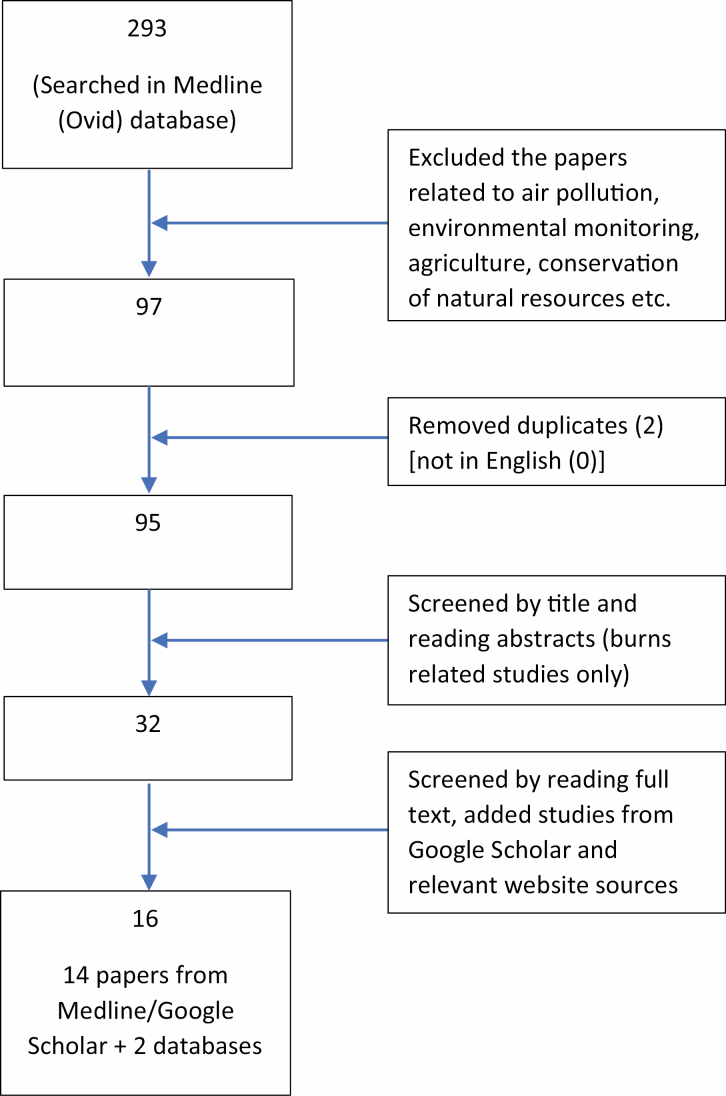
Literature search results and selection processes

A total of 293 studies were retrieved from the MEDLINE and google scholar databases. Ninety five papers from Nepal were identified after screening by language reported, duplication, and title (Figure 1). Abstracts of the 95 papers were screened and 32 papers specific to burns in Nepal were identified. Of these papers, 14 studies were identified as potentially relevant for estimating burn-related resource or cost and were reviewed in full.^12–25^Table 1 reports the key characteristics of these studies.

**Table 1.** Summary of reviewed studies and databases

Author and year	Setting	Location	Indication	Study type	Sample size (male/female)	Reported HCRU
Pujji et al 2019^18^	Kritipur Hospital/tertiary	Kathmandu	Burns	Retrospective	76 (23/53)	LOS Distance
Davé et al 2018^17^	Multi-country (including Nepal)	15 districts	Burns	SOSAS Survey	171 (burns) (98/74)	HCP contact*
Karki et al 2018^19^	Kritipur Hospital/tertiary	Kathmandu	Burns	Retrospective	567 (271/296)	LOS Surgeries
Cai et al 2017^20^	Kritipur Hospital/tertiary	Kathmandu	Burns	Modeled	na	Multiple (see text for details)
Tripathee and Basnet 2017^12^	Kritipur Hospital/tertiary	Kathmandu	Burns	Retrospective	284 (126/158)	LOS
Gupta et al 2015^14^	National	15 districts	Burns	SOSAS Survey	2695 (1434/1261)	NRR
Sharma et al 2015^13^	Bir Hospital/tertiary	Kathmandu	Burns	Retrospective	819 (428/391)	NRR
Rai et al 2015^21^	Kritipur Hospital/tertiary	Kathmandu	Burns	Retrospective	78 (32/46)	LOS Surgeries
Ghimere et al 2011^16^	Community	Dharan	Injuries	Survey	7063	Direct/indirect per patient cost*
Dahal and Paudel 2010^22^	Bir Hospital/tertiary	Kathmandu	Burns	Retrospective	100 (44/56)	LOS
Joshi et al 2009^15^	ED/health centers	Kathmandu Bhaktapur	Injuries	Prospective	505 (367/138)	Investigations* Drugs* Hospital costs*
Chalise et al 2008^23^	Nepal Medical College/secondary	Kathmandu	Burns	Retrospective	50 (29/21)	LOS
Shrestha 2006^24^	Patan Hospital/secondary	Lalitpur	Burns	Prospective	22 (9/13)	LOS
Liu et al 1998^25^	Western Regional Hospital/secondary	Pokhara	Burns	Prospective	237 (112/125)	LOS Surgeries
NMOH 2017^8^	All districts	Nationwide	All indications	Admissions statistics	na	NRR
GDB database^7^	na	Nationwide	Burns	Collated data	na	NRR
CHOICE database^9^	na	Nationwide	General population	Collated data	na	Bed day^†^ OP visit^†^ HCP contact^†^

*Healthcare resource and/or cost related to “injury” (not specific to burn).

^†^ Nonspecific healthcare unit costs.

*CHOICE*, choosing interventions that are cost-effective; *ED*, Emergency Department; *GBD*, global burden of disease; *HCP*, healthcare professional; *LOS*, length of hospital stay; *na*, not applicable; *NMOH*, Nepalese Ministry of Health; *NRR*, no resource reported; *OP*, outpatient; *SOSAS*, Surgeons Oversees Assessment of Surgical Needs; *WHO*, World Health Organization.

None of the studies reported any form of cost of illness analysis. Most of the research focused on the epidemiology and etiology of burns with only limited data relevant to the estimate of burns related resource. Two of the studies proved to have no information on resource use or cost.^13,14^ Two of the studies reported burns within the broader context of injury with only limited reference to burns and no data that were specific to burn-specific resource use or cost.^15,16^ One study reported cross-country surveys (including Nepal) on care seeking behavior in burns patients with no quantifiable data on resource.^17^ And one of the studies was a review of the epidemiological data and referenced a number of the other retrieved studies.^12^ The remaining eight studies provided some (although very limited) estimates of either resource use or expenditure^18–25^ with only one study reporting detailed costs (in a specialist cohort).^20^

None of the studies reported cost of admissions, treatment, or surgery. The only commonly reported resource metrics were length of stay for inpatient admissions (LOS) and number of surgeries. The eight studies reporting resource use or expenditure were all hospital-based and all reported the treatment of acute burns. Six studies were conducted in tertiary hospitals^18–22^ and three studies in secondary hospitals.^23–25^ Of the nine studies, seven reported on hospitals in the Kathmandu region. The majority of the hospital-based studies were retrospective data reviews (7 of 9). Three studies reported both mean LOS and number of surgical procedures in patients admitted to hospital with acute burns^19,21,25^ (one of these studies also reported blood transfusions following surgery). The remainder reported LOS only^22–24^ and LOS alongside distance traveled to the hospital.^18^ Distance traveled is often referenced as a measure of geographical service availability/provision but also as an indication of possible transport costs that would be incurred.

No information on cost of admissions was provided, only estimates of mean and total LOS. Mean LOS at secondary hospitals ranged from 13 to 16 days (total range 1–76 days)^23,25^ and at tertiary hospitals from 13 to 60 days (total range 1–124 days).^18,19,21,22^ Note that a proportion of patients in tertiary/specialist hospitalists were transferred from other hospitals, so this is not the total incident LOS. LOS was not broken down by severity or type of burn.

No information of cost of surgery was provided in any of the studies only number and percent of surgeries conducted. The percentage of patients who underwent surgery was reported for both secondary and tertiary hospital settings and included debridement, excision, skin grafting, and amputations. Secondary hospital surgery rate was reported at 52%.^25^ Tertiary hospital surgery rates were reported at 77 and 68%.^19,21^ One study additionally reported that 33% of patients required blood transfusion during skin graft surgery.^21^ The difference in rates across the settings likely reflects the degree of specialty of the hospitals.

Only one study reported detailed account of burn-specific expenditure and this was in the context of the setting up of Nepal’s first skin bank.^20^ The study provides information about cost per item for equipment and materials required for skin grafting alongside medical consumables and staff time. This is the only study which provides detail on the cost of medical consumables for burns treatment (including gauze, dressing, antibiotics, and pain medication) but the resource is specifically within the context of a graft population. Costs were categorized into start-up costs, consumable costs per donation (ie, each graft), and yearly costs of running the service. Total consumable costs were estimated in US$ at $118.80 per donation, with 75% of cost attributable to blood testing (including Hep C and HIV test kits), 12% to surgical consumables, 10% to dressings and lubricant, and around 3% to antibiotics. The rest of the cost comprised administrative necessities such as consent forms and certifications. Rolling costs (including staff costs and implementation of donation awareness programmes) were reported by year from initiation with a start-up cost of US$35,500 (no medical staff costs included) and year on year costs of around US$22,000 (of which around 25% was attributable to staff costs). Note that no skin grafts were conducted in year 1 (hence no staff costs).^20^

The GBD database was reviewed for Nepalese-specific burn data.^7^ Reported statistics and the NMOH source data were reviewed to check for relevant data.^7,8^ The GBD reported total burns and the NMOH database referenced reported numbers hospitalized due to burns. The relevant NMOH report was reviewed in detail in order to check whether any average admission cost could be estimated; however, no cost or expenditure data were reported.^8^

Our final search covered the WHO CHOICE database.^9^ The database did not provide any burn-specific estimates of resource or expenditure but did provide recommended costs in Nepalese Rupees (NPR) and international dollars (I$) to apply for bed days and healthcare visits. Reported cost for hospital bed days were NPR 264, I$18 (secondary) and NPR 361, I$24 (tertiary), for outpatient visits NPR 76, I$5 (secondary) and NPR 113, I$8 (tertiary), with health center visits estimated between NPR 94 and NPR 102 (I$6.33 to I$6.83) for a 20-minute visit (cost depending on population cover)^9^ (note that these unit cost reflect 2005 NPR and I$ so should be considered indicative at best).

## DISCUSSION

Our study highlights an almost complete lack of cost-focused burns research in Nepal. We did not find any studies which attempted to quantify the full burden and cost of burn care in Nepal or any part of the burn care pathway. The only study that reported a cost-based analysis was conducted in an extremely specific population and setting and is not generalizable to the broader burns population.

The published studies in Nepal mainly reported the etiology, types of burns, total body surface area affected by burn, and outcomes of burns treatment, including mortality.^18,19^ Economic markers were reported on a very limited scale and were restricted to top-line resource measures of LOS and likelihood of surgery.^18,19,21,22^ No studies reported any measure of long-term or rehabilitation-based resource use or expenditure.

The two studies that did report global cost studies were limited to cost of injuries and did not contain any burn-specific data.^15,16^ These studies were not helpful in answering our study question because there are recognized differences in the cost of burns care compared with costs of the care of more general injuries.^26,27^

Only one paper reported items and costs related to burn treatment in Nepal.^20^ This paper presented start-up costs for equipment and costs of items required for burn treatment such as dressings, surgical consumables, antibiotics, and different disease testing kits. However, the paper had a purpose of establishing a skin bank for burn treatment and the key focus of the paper was to report the feasibility and challenges of creating this in Nepal. Although important in the context of the paper, the study does not allow for an estimate of the general cost of burns.

Official data by the Nepalese Ministry of Health and Population did not include any information on costs. The cost data from the WHO CHOICE study are included here in the absence of any other cost data as they could usefully be combined with LOS estimates to provide a crude cost of inpatient admission or with HCP contacts to provide a crude cost of outpatient or health center care. However, the approach would allow for only a very global estimate of costs based on broad LOS or estimated healthcare contacts and would not help in costing out surgeries, dressings, or drug treatments (these are not covered in top-line estimates of inpatient or outpatient costs).

Previous studies have summarized and reported the costs of burns in other countries.^4,28–33^ These studies highlight the disparities in approach and the challenges in delivery but also the importance of such estimates in focusing political and funding attention on burns.^4,32^ The studies conducted in other LMICs have covered a range of methodologies including bottom-up costing at a hospital level with costs limited to payer or government-based expenditure^30^ and survey-based analyses covering wide geographies and encompassing household expenditures.^33^ The extreme lack of data in Nepal provides a blank slate for future research activities but the hospital-based methods may provide a more time manageable and targeted option for initial research.

It is clear from the review that while burns are a serious public health problem in Nepal, the current status of cost-focused research in Nepal is extremely limited. The absence of evidence for Nepal should motivate additional studies to generate evidence that could help in making a compelling case for political attention and funding, by quantifying the true cost of burns in Nepal. In such a situation, conducting manageable research collecting data from primary sources appears a sensible way forward. Our recommendation would be to focus on the cost of tertiary care in the first phase. While not providing a complete estimate of burn burden in Nepal, severe burn injuries require the highest level of intensive treatment^12^ and identification of these costs could be a first step toward emphasizing the political importance of prevention and management programmes.

This is a structured review where we tried to identify different cost components of burns treatment to estimate national level cost burden of burns. We found almost no evidence of cost-focused research in Nepal. Robust primary research could help fill the gaps in estimation of economic burden of burns. However, a robust estimation of national level economic burden of burns is some way away.
